# Small bowel T-cell lymphoma with perforation in the HIV/AIDS context, a rare case report

**DOI:** 10.1016/j.ijscr.2025.111087

**Published:** 2025-02-27

**Authors:** Musa Machibya, Bhavish Damji, Shabbir Adamjee, Willbroad Kyejo, Aidan Njau, Caroline Ngimba

**Affiliations:** a Department of General Surgery, The Aga Khan University, P. O Box 38129, Dar Es Salaam, Tanzania; b Department of Family Medicine, The Aga Khan University, P. O Box 38129, Dar Es Salaam, Tanzania; c Department of Pathology, The Aga Khan Hospital, P. O Box 2289, Dar Es Salaam, Tanzania

**Keywords:** Small bowel lymphoma, Bowel perforation, HIV/AIDS, Case report, T cell lymphoma

## Abstract

Introduction and Importance.

Small bowel lymphomas, which represent <1 % of gastrointestinal cancers, are most found in the ileum due to its high concentration of gut-associated lymphoid tissue. T-cell lymphomas of the small bowel are particularly rare. While increased risk is noted in conditions like celiac disease and immunodeficiency, their occurrence in HIV/AIDS patients is uncommon and poorly understood. The rare complication of gastrointestinal perforation in these cases complicates diagnosis and management.

**Case presentation:**

42-year-old woman with HIV/AIDS, on antiretroviral therapy for 4 years, presented with a 1-month history of progressively worsening abdominal pain, intermittent fevers, weight loss, and a productive cough. On physical examination, she was cachectic, febrile, and had generalized abdominal tenderness with signs of peritonism. Laboratory investigations revealed anemia, elevated CRP, and ESR, with a CD4 count of 441 cells/mcL. Imaging studies, including abdominal CT, showed free air and fluid suggestive of a perforated viscus. The patient underwent emergency laparotomy, which revealed multiple bowel perforations. Resection was performed, and histopathology confirmed small bowel T-cell lymphoma (anaplastic large cell lymphoma). Despite intensive postoperative care, the patient died on the fourth postoperative day.

**Clinical discussion:**

Small bowel T-cell lymphoma is rare, especially in HIV/AIDS patients, and poses significant diagnostic challenges. Its presentation is often nonspecific, and perforation is a serious complication. While chemotherapy and surgery are key treatments, T-cell lymphomas are more resistant to therapy, leading to a poor prognosis, particularly when complicated by perforation.

**Conclusion:**

This case highlights the rarity and complexity of small bowel T-cell lymphoma in HIV/AIDS, compounded by gastrointestinal perforation. Early diagnosis, advanced imaging, and multidisciplinary management are essential for improving outcomes. Further research is needed to optimize treatment strategies for this challenging case.

## Highlights

• Rare presentation: small bowel T-cell lymphoma diagnosed following perforated viscus in HIV/AIDS patient.
• Diagnostic challenge: delayed recognition due to nonspecific symptoms in an immunocompromised individual.
• Complex management: successful emergency exploratory laparotomy despite advanced disease and poor prognosis.

## 1 Introduction

Lymphomas are proliferations of lymphoid cells commonly found in the lymph node or extra nodal tissues. Small bowel lymphomas constitute a subset of gastrointestinal malignancies, representing <1 % of cases, commonly found in the ileum, where there is the greatest concentration of gut-associated lymphoid tissues [1].

Primary small bowel neoplasms are rare, constituting 1–5 % of all gastrointestinal cancers. Lymphomas account for 1–4 % of gastrointestinal neoplasms, with 30 % involving the small intestine. Most primary gastrointestinal lymphomas are of B-cell origin, with <10 % being primary T-cell non-Hodgkin lymphoma [2,3].

An increased risk for development of primary small bowel lymphomas was reported in patients with celiac disease and immunodeficient states like HIV/AIDS [4,5].

Epidemiological studies have reported that the incidence of T-cell lymphoma in the context of HIV/AIDS-related non-Hodgkin lymphoma, with unclear pathogenesis, ranges from 1.4 % to 3 % [6,7].

This condition typically presents with symptoms such as abdominal pain, weight loss, nausea, vomiting, and altered bowel habits. Perforation is a common complication, occurring in up to 25 % of cases, along with other reported complications such as bowel obstruction. Fever, though rare, may indicate systemic involvement. Grossly, lesions are often large (>5 cm) and may extend beneath the mucosal layer. Histopathologically, diffuse infiltration of the intestinal wall is frequently observed [8,9].

The treatment of small bowel lymphoma is debated. While surgery, chemotherapy, and radiation were traditionally used, asymptomatic cases are often chemo-responsive and may not require surgery. B-cell lymphomas are more chemo-sensitive with high remission rates, whereas T-cell lymphomas, being more resistant, may progress to obstruction or perforation if not surgically resected [8].

Regardless of cell type, resection is indicated at any onset of symptoms because progression to life-threatening hemorrhage or perforation portends a dismal prognosis. Five-year survival of 50 % to 60 % can be expected and is dictated by response to systemic therapy rather than by the success of surgical resection [8].

This study aims to address key research gaps related to the rare occurrence of small bowel T-cell lymphoma in the context of HIV/AIDS, focusing on diagnostic and management challenges, the impact of HIV-associated immunodeficiency on lymphoma behavior, and complications like gastrointestinal perforation. In this report, we present a detailed case of small bowel T-cell lymphoma in a patient with HIV/AIDS, whose clinical course was marked by the rare occurrence of gastrointestinal perforation. It will provide valuable data on prognosis, survival outcomes, and the need for early detection and personalized management strategies in this unique patient population.

This case report has been reported in line with Surgical Case Report guidelines (SCARE) [10].

## 2 Case presentation

A 42-year-old woman with known HIV/AIDS on Tenofovir, Lamivudine and Dolutegravir for the past 4 years, presented with the complaints of abdominal pain for 1 month, that was of gradual onset, with no specific periodicity or relation to meals. She described the pain to be dull, generalized throughout the abdomen, more marked in the periumbilical region. The pain gradually worsened with time and was not relieved by oral analgesics. She reported no history of nausea, vomiting or change in bowel habits. There was no history of tarry black or pale stools, or blood in stools.

The patient also reported subjective fevers for the past several months that were intermittent, associated with chills and proceeded by drenching sweats, more marked during the evenings. The fevers were occasionally relieved by oral paracetamol.

Reported to have a 4-week history of productive cough, yielding 2–3 tablespoons of thick yellow sputum, without hemoptysis, chest pain, or dyspnea. She also reported unintentional weight loss over the past few months. The patient has a history of multiple prior admissions for infectious conditions, including recent treatment for bronchopneumonia complicated by bronchiectasis, 5 days prior to the current admission.

The patient is a mother of two children, worked at a bank and had been living alone, with only occasional visits from relatives. The patient had history of regular indulgence of alcohol, having calculated 41 units of alcohol per week for several years, but had abstained after the onset of her illness. She is a non-smoker. Family and social support may have been regarded to be insufficient during the course of her illness. Thus, the adherence to her ART regimen was not well ascertained.

On physical assessment, the patient was ill-looking, cachectic, alert, and afebrile, found to be pale with a tinge of jaundice. No peripheral lymph nodes were palpable. No peripheral edema noted. Vital signs were stable: Blood pressure of 102/74 mmHg, Mean arterial pressure of 84 mmHg, pulse rate of 20/min, respiratory rate 20/min, Spo2 of 98 % on room air.

Per abdominal examination revealed the slightly scaphoid, moved on respiration, with inverted umbilicus. A longitudinal median scar was noted. There was generalized tenderness on superficial and deep palpation, with guarding, rigidity and rebound tenderness. No organomegaly or masses were palpated. Bowel sounds were normal. Genital exam was unremarkable. Respiratory examination revealed bilateral coarse crackles with bilateral equal air entry. Other systems were essentially unremarkable.

**Fig. 1:**
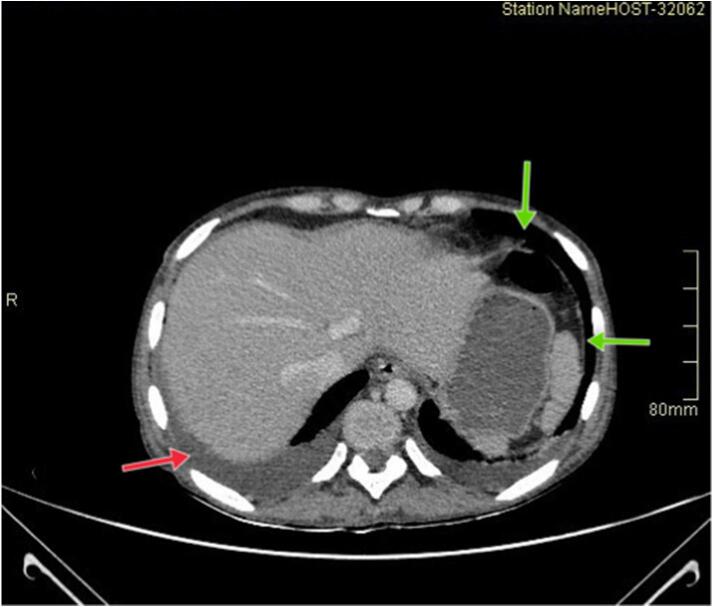
Axial CT scan image of the abdomen with significant free air in the peritoneal cavity consistent with pneumoperitoneum (green arrows), with fluid collection (red arrow). (For interpretation of the references to color in this figure legend, the reader is referred to the web version of this article.)

The CT findings demonstrated pneumoperitoneum with free intraperitoneal fluid, likely secondary to a perforated viscus (Fig. 1).

The laboratory results indicated anemia with a hemoglobin level of 7.2 g/dL, normal white cell counts as well as signs of inflammation, with an elevated ESR of 78 mm/h and CRP level of 298.7 mg/L. Deranged liver function tests. The CD4 count was 441 cells/mcL, and the viral load was 66 copies/mL.

Following the impression of a perforated viscus, the patient was kept on nil per mouth, given intravenous fluids, antibiotics and was prepared for an emergency exploratory laparotomy. Intraoperative had a gush of air observed upon opening the peritoneum. The abdomen was grossly contaminated with bilious contents. Along the bowel, four perforations were noted, of which two were 3 × 2 cm, and the other two 2 × 1 cm. (Video 1) Mesenteric lymph nodes were grossly enlarged. Hepatomegaly was noted. Peritoneal deposits. 30 cm from duodenojejunal junction, ileus loop was exteriorized, along with distal end of a resected small bowel segment. Three perforations were wedge resected. Bowel lesion and lymph node were resected and taken for histopathological evaluation.

**Fig. 2:**
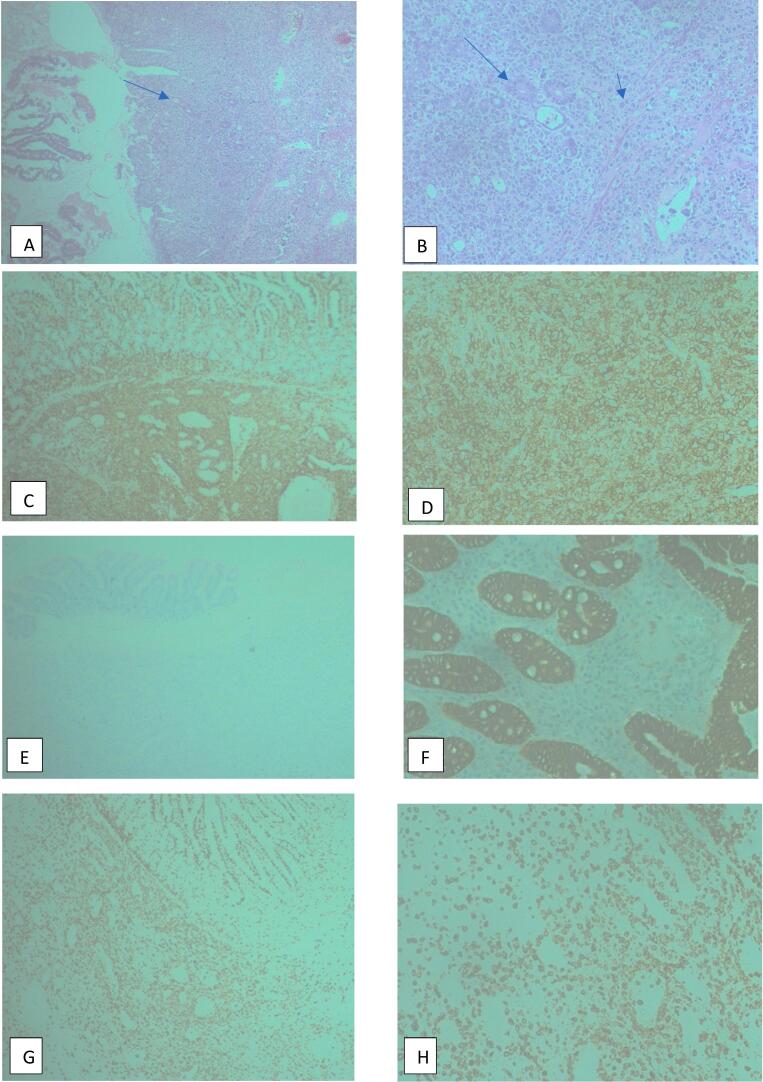
H&E sections (A-B) show ileal mucosa glands (arrow) replaced by diffuse infiltrates of neoplastic cells (arrowhead), which are large, round, with irregular nuclei and prominent nucleoli, some displaying bizarre, enlarged nuclei, while immunohistochemistry for CD45 (C-D) and CD3 (E-F) demonstrate diffuse positivity in the neoplastic cells, confirming a T-cell lymphoid origin, with negative CD3 staining in G and negative Pancytokeratin staining in H, where Pancytokeratin is positive in mucosal glands (internal positive control).nucleoli, some displaying bizarre, enlarged nuclei, while immunohistochemistry for CD45 (C-D), with negative CD3 staining in E and negative Pancytokeratin staining in F, where Pancytokeratin is positive in mucosal glands (internal positive control) and CD3 (G-H) demonstrate diffuse positivity in the neoplastic cells, confirming a T-cell lymphoid origin.

Report from histopathological examination revealed the sections from the ileum showing the fragments of tissue composed of mucosa glands with areas of ulcerations and perforation. There was diffuse infiltration of the wall by neoplastic cells which are large with irregular nucleus, and prominent nucleoli. The histological features were suggestive of high-grade lymphoma. Immunohistochemistry results showed: Pan cytokeratin negative, CD3 positive, CD45 strong and diffuse positive, CD20 negative. The final diagnosis was thus concluded to be small bowel T-cell lymphoma (anaplastic large cell lymphoma) (Fig. 2).

The patient was kept under close observation in the intensive care unit. Unfortunately, the patient died on the fourth postoperative day due to multiorgan failure with the course established being sepsis.

## 3 Discussion

Lymphomas originating in the digestive tract are rare, accounting for a small percentage of gastrointestinal malignancies. These lymphomas arise from the mucosa-associated lymphoid tissue (MALT), which is essential for immune function in the gastrointestinal tract [8]. In sub-Saharan countries like Tanzania, limited access to diagnostic tools such as immunohistochemistry leads to underreporting and challenges in the accurate detection of lymphoma subtypes, which in turn hinders treatment strategies.

Intestinal T-cell lymphoma, making up <10 % of primary GIT NHLs, is more aggressive than B-cell lymphoma, which is more common (6:1 ratio). T-cell lymphoma has a 23.8 % five-year survival rate, compared to higher rates for B-cell lymphomas [3,11,12]. Explaining the rarely occurrence in our case.

As stated by Dawson et al., the diagnosis of gastrointestinal lymphoma should fulfill five criteria: (1) no palpable superficial lymphadenopathy; (2) no enlarged mediastinal lymph nodes; (3) normal total and differential white blood cell counts; (4) a predominance of bowel lesions observed during laparotomy, with lymph nodes primarily affected in the surrounding area; and (5) no involvement of the liver and spleen. The small intestine is the second most affected site in primary GI lymphoma, following the stomach [13]. These findings are consistent with our case, where the diagnosis aligns with the absence of systemic lymphadenopathy and predominant bowel involvement, further supporting the diagnosis of primary gastrointestinal lymphoma.

Clinical presentation of gastrointestinal lymphoma is often nonspecific and can include abdominal discomfort, loss of appetite, nausea, vomiting episodes, signs of peritonism due to perforation and B symptoms such as fever and weight loss [8].

The literature reviews conducted by Tian Y et al., Yanagi M et al., and Hussain M et al. all describe the rare complication of small bowel perforation in cases of small bowel T-cell lymphoma. These findings are consistent with the clinical presentation observed in our patient, who presented late with a perforated small bowel as a complication of the lymphoma [[14], [15], [16]].

World Health Organization classified intestinal T-cell lymphomas into four subtypes: enteropathy-associated T-cell lymphoma (EATL), monomorphic epitheliotropic intestinal T-cell lymphoma (MEITL), indolent T-cell lymphoproliferative disorder, and intestinal T-cell lymphoma, not otherwise specified (NOS) [17]. Other common subtypes include peripheral T-cell lymphoma (PTCL) and NK/T-cell lymphoma (NK/TL), with anaplastic large cell lymphoma being a rare but more aggressive subtype [18].

Given this, our patient underwent serology for celiac disease to rule out EATL as the most common differential for small bowel T cell lymphoma postoperatively, which came out negative. Histopathological evaluation revealed ALK positivity, consistent with the anaplastic large cell lymphoma subtype, which is rare and more aggressive compared to the other subtypes.

Various factors such as bacterial and viral infections, immune deficiencies like HIV/AIDS, and celiac disease are believed to contribute to their development [8].

In the context of HIV/AIDS, individuals typically have a significantly higher risk of developing peripheral T-cell lymphoma (PTCL), with the risk being 24 times greater than in the general population [19]. Most cases occur in males (around 80 %), with a median age of diagnosis of approximately 38 years. The typical HIV-related PTCL patient has a low CD4 count (around 150 cells/mm^3^) and a high viral load (around 300,000 copies/mm^3^). The most common PTCL subtype in HIV patients is PTCL-NOS, followed by anaplastic large cell lymphoma (ALCL), NK/T-cell lymphoma, and angioimmunoblastic T-cell lymphoma (AITL) [20].

In contrast, our patient is a middle-aged female with a relatively higher CD4 count of 441 cells/mm^3^ and a low viral load of 66 copies/mm^3^, indicating a better-controlled HIV infection and relatively preserved immune function. Despite these factors, the patient has been histologically diagnosed with anaplastic large cell lymphoma (ALCL), which, although less common than PTCL-NOS, is still a recognized subtype of PTCL in HIV-positive individuals. This highlights that PTCL can affect patients even with higher immune function and better viral control.

Imaging studies, such as barium studies and CT scans, may reveal masses or areas of infiltration; however, endoscopy with biopsy remains the gold standard for diagnosis [21]. Pneumoperitoneum can be observed in the setting of perforation, as was evident in our patient.

Treatment strategies for gastrointestinal lymphomas depend on the histopathological subtype and disease stage. Chemotherapy with cyclophosphamide, doxorubicin, vincristine, and prednisolone (CHOP) is a mainstay, with regimens tailored to the specific lymphoma type. Surgical resection may be considered in select cases, particularly for low-grade B-cell lymphomas of the small intestine [22,23].

The β-catenin-LINC00183-miR-371b-5p-Smad2/LEF1 axis promotes T-cell lymphoma progression and chemoresistance, as seen in T-cell lymphoblastic lymphoma [24]. This mechanism may also be relevant to small bowel T-cell lymphoma (SBTCL) in HIV/AIDS patients, highlighting similar challenges in treatment response and chemoresistance. Understanding these molecular pathways could improve management strategies for both conditions.

Complications such as perforation can occur, particularly during chemotherapy, occurring in about 9 % of cases, with a median time to perforation of roughly 46 days, but do not necessarily impact overall prognosis [25,26].

Our patient underwent an emergency exploratory laparotomy due to bowel perforation. Four perforations along the bowel were identified. An ileus loop was exteriorized at 30 cm from the duodenojejunal junction, along with the distal end of a resected small bowel segment. Three perforations were wedge resected, and the bowel lesion, along with the affected lymph node, was resected. The patient was scheduled to undergo chemotherapy; unfortunately, the patient demised before the intervention post-operatively. Diagnostic staging was done retrospectively using CT scan findings according to the Ann Arbor Staging System for ALCL, which revealed lymph nodes above and below the diaphragm with disseminated disease involving the mesenteric lymph nodes and with peritoneal deposits consistent with stage IV disease.

## 4 Conclusion

Small bowel T-cell lymphoma should be considered in the differential diagnosis of abdominal symptoms in HIV/AIDS patients, especially in cases of gastrointestinal perforation. Due to its rarity and low incidence, intestinal perforation caused by lymphoma is often overlooked and can be difficult to distinguish from conditions like Crohn's disease, intestinal tuberculosis, and intestinal typhoid. In patients presenting with abdominal pain, fever, and perforation particularly when multiple ulcers are observed intraoperatively or during evaluation, the possibility of lymphoma should be considered to prevent misdiagnosis. Early recognition and timely intervention are crucial for optimizing outcomes in these rare but potentially life-threatening cases. Therefore, gastrointestinal surgeons, must improve their understanding of this rare condition. Further research is needed to determine the best management strategies for small bowel lymphomas in the context of HIV/AIDS.

The following is the supplementary data related to this article.

Video S1 Imaging reveals lymphoma nodules and perforations in the ileum. Video S1

## CRediT authorship contribution statement

1. Musa Machibya: Study conception, production of initial article, collection of data, final
2. manuscript writing.
3. Caroline Ngimba: Study conception, revision of the article, and proofreading.
4. Bhavish Damji: Production of initial article and collection of data.
5. Shabbir Adamjee: Revision of the article and proofreading.
6. Willbroad kyejo: Study conception, revision of the article, and proofreading.
7. Aidan Njau: Study conception, revision of the article, and proofreading.

## Informed consent

Written informed consent was obtained from the patient husband for the anonymized information to be published in this article.

## Ethics approval

Our institution does not require ethical approval for reporting individual cases report.

## Guarantor

Dr. Musa Machibya

## Funding

The author(s) received no financial support for the research, authorship, and/or publication of this article.

## Declaration of competing interest

The author(s) declared no potential conflicts of interest with respect to the research, authorship, and/or publication of this article.
